# Prediction of Treatment Outcome with Inflammatory Biomarkers after 2 Months of Therapy in Pulmonary Tuberculosis Patients: Preliminary Results

**DOI:** 10.3390/pathogens10070789

**Published:** 2021-06-22

**Authors:** Simona Stefanescu, Relu Cocoș, Adina Turcu-Stiolica, Elena-Silvia Shelby, Marius Matei, Mihaela-Simona Subtirelu, Andreea-Daniela Meca, Elena Camelia Stanciulescu, Stefana Oana Popescu, Viorel Biciusca, Catalina-Gabriela Pisoschi

**Affiliations:** 1 Clinical Analysis Laboratory, Clinical Emergency County Hospital Craiova, 200642 Craiova, Romania; simonastefanescubio@gmail.com; 2 Department of Medical Genetics, University of Medicine and Pharmacy “Carol Davila”, 020032 Bucharest, Romania; 3 Institute of Pneumophtisiology “Marius Nasta”, 050159 Bucharest, Romania; 4 Department of Pharmacoeconomics, University of Medicine and Pharmacy of Craiova, 200349 Craiova, Romania; adina.turcu@gmail.com (A.T.-S.); mihaela.subtirelu@yahoo.com (M.-S.S.); 5 Scientific Research Nucleus, Dr. Nicolae Robanescu National Clinical Centre for Children’s Neurorecovery, 041408 Bucharest, Romania; silviajdx@yahoo.com; 6 Department of Histology, University of Medicine and Pharmacy of Craiova, 200349 Craiova, Romania; mariusmatei44@yahoo.com; 7 Department of Pharmacology, University of Medicine and Pharmacy of Craiova, 200349 Craiova, Romania; andreea_mdc@yahoo.com; 8 Department of Biochemistry, University of Medicine and Pharmacy of Craiova, 200349 Craiova, Romania; camiparsot@yahoo.com (E.C.S.); stoapo@yahoo.com (S.O.P.); c_pisoschi@yahoo.com (C.-G.P.); 9 Department of Internal Medicine, University of Medicine and Pharmacy of Craiova, 200349 Craiova, Romania; biciuscaviorel@gmail.com

**Keywords:** pulmonary tuberculosis, C-reactive protein, white blood cells, IP-10, LL-37, CAR, NAR

## Abstract

Pro-inflammatory mediators play an important role in the pathogenesis of pulmonary tuberculosis. Consecutively, 26 pulmonary tuberculosis patients were enrolled in our study based on the exclusion criteria. We have used Spearman’s correlation analysis, hierarchical clustering and regression modelling to evaluate the association of 11 biomarkers with culture status after antituberculosis treatment. The results of our study demonstrated that six inflammatory biomarkers of 11, C-reactive protein (CRP), white blood cells (WBC), neutrophils, interferon gamma inducible protein 10, C-reactive protein (CRP) to albumin ratio (CAR) and neutrophil to albumin ratio (NAR), were significantly associated with culture negativity. The predictive ability of a composite model of seven biomarkers was superior to that of any single biomarker based on area under the receiver operating characteristic curve (AUC) analysis, indicating an excellent prediction efficacy (AUC:0.892; 95% CI:0.732-1.0). We also found that the highest significant trends and lower levels of CRP and IP-10 were observed in the two-month treated tuberculosis (TB) patients. We believe that our study may be valuable in providing preliminary results for an additional strategy in monitoring and management of the clinical outcome of pulmonary tuberculosis. Using a panel of predictors added a superior value in predicting culture status after anti-TB therapy.

## 1. Introduction

Tuberculosis (TB) remains one of the leading causes of mortality worldwide with more than 1.6 million deaths annually reported [1,2,3]. The newly diagnosed patients with *Mycobacterium tuberculosis* (*Mtb*) commonly receive the first-line therapy consisting of four-drug regimen, isoniazid (H), rifampicin (R), pyrazinamide (Z), and ethambutol (E) for two months, followed by a four-month continuation phase [4,5]. Currently, sputum culture conversion (SCC) or smear microscopy (SSM) are used as standard measures for monitoring of the treatment response together with radiological evaluation [6].

During TB infection, proinflammatory mediators play an important role in the pathogenesis by initiating, intensifying, and maintaining the inflammatory process, while other inhibitors diminish it as a part of the protection and recovery process [7]. The cellular immune response associated with *Mtb* is enabled by activation of macrophages and lymphocytes that in turn secrete cytokines and other factors which determine the disease severity [8].

In recent years, a milieu of inflammatory factors, cytokines and chemokines have been found to be useful in immune diagnosis of TB and monitoring the efficacy of therapy, including C-reactive protein (CRP), interferon-γ [IFN-γ], interleukin-6 [IL-6], tumor necrosis factor—α [TNF-α] or chemokine interferon-gamma inducible protein-10 (IP-10) [9,10,11,12]

Some hematological inflammatory markers of TB patients, such as white blood cell (WBC) counts, mean platelet volume (MPV), neutrophil counts (NEU) have provided enlightening insights into monitoring the outcomes of tuberculosis treatment [13,14,15].

IP-10 has been widely evaluated as a biomarker for TB and it has been reported to be expressed at 100-fold higher levels than IFN-γ with a greater detection reproducibility [12]. IP-10 is secreted by several cell types such as monocytes or neutrophils, and induce the chemoattraction of other cells including monocytes and T cells at inflammatory sites [16].

Additionally, another studied serum biomarker in the management of the inflammatory process is human cathelicidin (LL-37 peptide), the C-terminal sequence of human antimicrobial peptide-18 (hCAP18) formed after extracellular cleavage, which exhibits an important role in the innate immune response against *Mtb* [17].

During *Mtb* infection, the expression of LL-37 is induced by the upregulation of vitamin D receptor as a result of the interaction of mycobacterial lipopeptide with Toll-like receptors (TLR) [18].

LL-37 maintains an equilibrium between pro-inflammatory and anti-inflammatory responses in order to eliminate pathogens by regulating the production of different chemokines and cytokines. Alteration of the production of LL-37 increases the susceptibility to infectious disease, including TB [19] with higher concentrations of LL-37 corresponding to an exaggerated immune activation and increased disease burden. Previous findings suggested that LL-37 can be used as a potential biomarker for the diagnosis of TB due to the increase of the plasma level in the compensatory response detected in patients with pulmonary tuberculosis (PTB) [20], but there is a limited number of studies that have investigated the LL-37 marker in combination with other pro-inflammatory biomarkers to delineate its potential predictive role in monitoring of treatment response in patients with TB.

C-reactive protein (CRP), an acute phase protein produced by hepatic cells following the stimulation induced by TNF-α, IL-1and IL-6, was found to be the most potent marker for diagnosis of active TB when compared to other inflammatory markers [21]. C-reactive protein is a non-specific inflammatory marker that has been proven useful in predicting inflammatory conditions, but with a relatively slow responsive kinetic profile that cannot accurately reflect the bacterial load, hindering its clinical use [5,22]. Reductions in CRP values after the two-month intensive phase treatment have been reported in several studies [23]. Positive correlations have been noted between the decrease in inflammatory markers, such as CRP and IP-10 levels, and the degree of lung involvement [24] and sputum culture conversion [25]. Moreover, the change in concentrations of various biomarkers after two months treatment, such as the concentrations of CRP and IP-10, have been associated with an improvement of the evolution of radiographic lung lesions [26], but assessment of the extent of radiographic lesions with treatment as a tool to evaluate disease activity was outside of the aim of the present study.

Albumin and globulin are the major constituents of human total serum proteins which play an important role in inflammation [27]. Albumin is a negative acute-phase protein that was found to decrease in response to inflammation in chronic infection [28], whereas high levels of globulin were reported in cancer or rheumatic diseases [29]. The C-reactive protein (CRP) to albumin ratio (CAR), albumin to globulin ratio (AGR), neutrophil to albumin ratio (NAR) are novel markers of systemic inflammation that have been investigated in patients with different conditions and their clinical importance has not yet been assessed in PTB [30,31,32]. Although these ratios have been investigated in many diseases, there is no study on the use of them in PTB.

Many studies have demonstrated that increased levels of inflammatory biomarkers are associated with poor outcomes in patients with PTB [33,34]. Sputum culture conversion evaluated at two months of anti-TB chemotherapy could be used to predict clinical outcome in TB as a biomarker of response to treatment [35,36]. As an alternative to sputum culture conversion, various single or combinations of blood-based biomarkers have been identified as having a predictive role in diagnosis and monitoring the efficacy of therapy [26].

After treatment initiation, the levels of the majority of biomarkers tend to decrease significantly, indicating a reduction of the severity of lung injury and a decline of the bacterial burden [37], which reflects, as many authors suggested, that a fine control of inflammatory response is essential for clinical status improvement.

Immune-based biomarkers could be predictive for PTB treatment with respect to responses before the initiation of treatment or monitoring follow-up therapeutic outcomes [38,39,40]. Although there have been different studies exploring single pro-inflammatory markers, the combination of our hematological and biochemical inflammatory biomarkers has not been yet intensively investigated.

Various inflammatory parameters can be combined into a multiple biomarker in order to significantly improve the prediction accuracy of the prognosis and the response to antibiotic chemotherapy [10,41].

This study hypothesized that a certain panel of inflammatory markers would have higher monitoring accuracy for anti-TB treatment efficacy than single markers considering they are non-specific inflammatory markers. We selected markers that previously appeared to be the most consistently associated with TB disease progression in combination with markers that have been poorly evaluated for management of TB treatment as single markers or in composite panel.

A potentially synergistic decline in the levels of a set of biomarkers after successful anti-tuberculosis therapy might be useful to monitor and evaluate the treatment response. In addition, immunological biomarkers should be validated in various geographical and ethnic settings due to other intrinsic and extrinsic factors that can act as a determinants of disease outcome.

Thus, to contribute to this field, the aim of our pilot prospective study was to assess the predictive role of a certain set of inflammatory biomarkers, either individually or in combinations for monitoring treatment response after the two-month intensive phase.

## 2. Results

Of 38 patients diagnosed with TB during the study period, 26 patients were included in the final analysis based on the exclusion criteria and received tuberculosis treatment at enrollment. Patients had a median age of 47.85 years and 23 (88%) were male. Additional demographic information can be found in Table 1. On chest radiography, the most common types of radiological lesions on admission were as follows: infiltrate opacities (n = 4), nodular opacities (n = 16), cavities opacities (n = 6) and pleural effusion (n = 2). Among the 26 patients who were finally diagnosed with PTB, the two-month chest X- ray showed improvement in 18 (69%) and no improvement or aggravation in 8 patients (31%). Our results have shown no significant correlation between the types of radiographic lesions and the values of pro-inflammatory markers.

All patients subjected to the drug susceptibility testing demonstrated no resistance to the first line anti-TB drugs.

All patients were tested culture positive at enrollment in the study (T0). Twenty patients (77%) had negative culture conversion after two months of TB treatment. The laboratory data are included in Table S1.

As shown in Table 2, we analyzed the differences between the values of biomarkers in patients that turned negative culture after the two months of treatment (n = 20, T2) versus the same patients before treatment initiation (T0). The treatment effect with negative sputum culture conversion status at two months was significantly associated with lower values at T2 for CRP, WBC, NEU, IP10 and CAR and NAR ratios, respectively. The NAR ratio was significantly lower in patients that turned negative culture in parallel with the decline in neutrophil levels (*p*-value = 0.0061), Table 2.

Serum levels of IP10 changed considerably between patients with positive culture (T0) that turned culture negative after 60 days of anti-microbial treatment (*p*-value = 0.0080), while serum levels of LL37 presented no statistical difference (*p*-value = 0.1918), as shown in Table 2.

**Figure 1:**
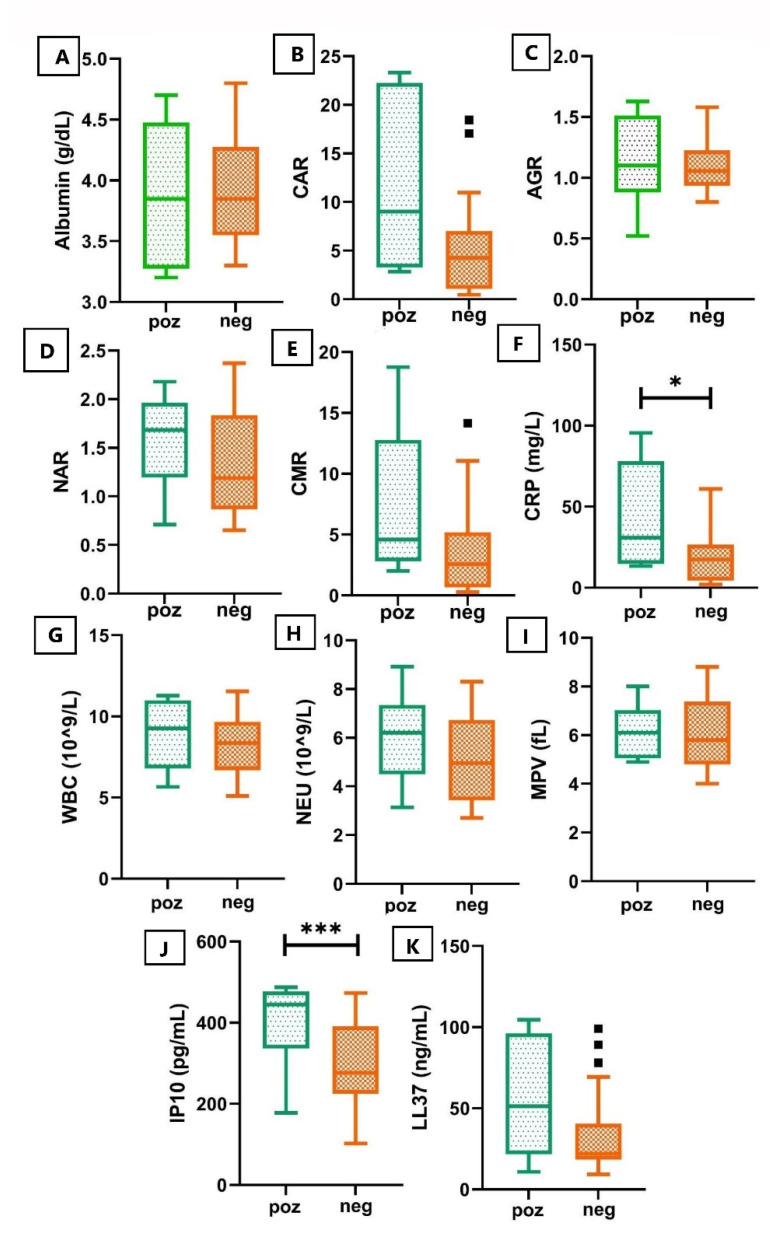
Comparative box and whisker plots for all biomarkers. Box-and-whisker plots representing median of Albumin (**A**), CRP (**F**), WBC (**G**), NEU (**H**), MPV (**I**), IP10 (**J**) and LL37 (**K**) levels and CAR (**B**), AGR (**C**), NAR (**D**) and CMR (**E**) ratios. Green box designates a boxplot of the values at therapy completion from values at the T2 time for the culture positive patients (n = 6). Orange box designates a boxplot of the values at therapy completion from values at the T2 time for the culture negative patients (n = 20). The horizontal line inside the box indicates the median value. Whiskers extend to the largest and smallest observed values within the box lengths. ■, outliers. The asterisks indicate that the difference between two groups is significant (*, *p* < 0.05; ***, *p* < 0.001).

When comparing the subtracted biomarkers’ median values or ratios from median values or ratios at T0 (valuesT2–valuesT0) amongst the patients with positive culture (n = 6) versus the patients with negative culture (n = 20) to better view the difference before treatment initiation, we observed only two biomarkers with significantly decreased levels, CRP, and IP-10, as illustrated in Table 3 and Figure 1. As shown in Table 3, no significant changes were observed for the rest of the biomarkers when comparing the subtracted values at therapy completion from values at the time of diagnosis of the patients with positive culture versus the patients with negative culture.

To further reveal the correlations among our biomarkers pairwise, Spearman correlation coefficients were calculated and ordered by hierarchical clustering to look for patterns. Based on the Spearman correlation analysis, the highest significant positive correlations (Spearman’s correlation coefficient > 0.8, *p*-value < 0.05) were found for CMR and CAR, CRP with CAR, CRP with CMR, NAR with NEU, WBC with NAR, and WBC with NEU, as shown in Table 4.

**Figure 2:**
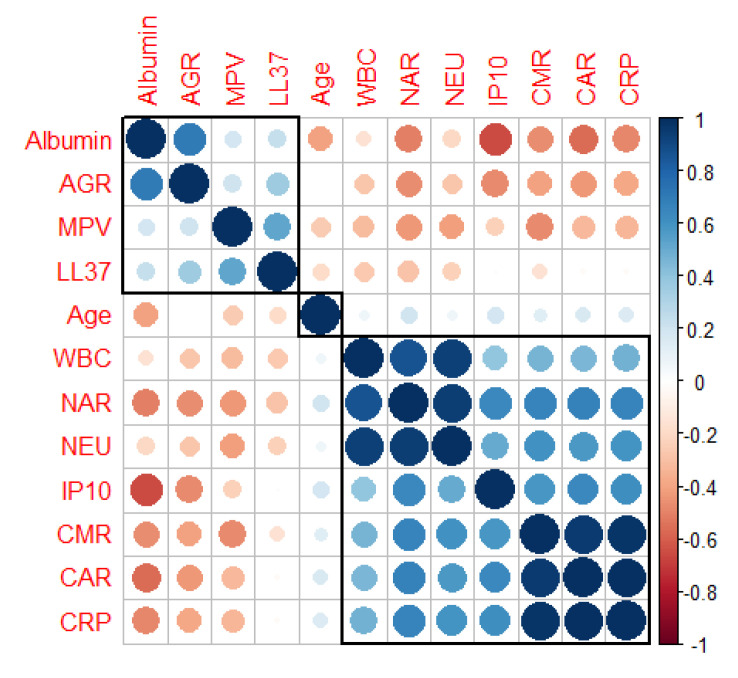
Correlogram with hierarchical clustering of covariates included in the logistic regression analysis. Positive and negative correlations are represented by blue and red dots. The sizes and the shades of the dots reflect the strengths of correlation between pairs of biomarkers and ratios. Colors range from bright blue (strong positive correlation; i.e., r = 1.0) to bright red (strong negative correlation; i.e., r = −1.0). Correlations are ordered by hierarchical clustering with clusters outline.

In addition, two clusters with smaller groups of closely related biomarkers were identified by hierarchical clustering analysis and visualized by correlogram, Figure 2.

**Figure 3:**
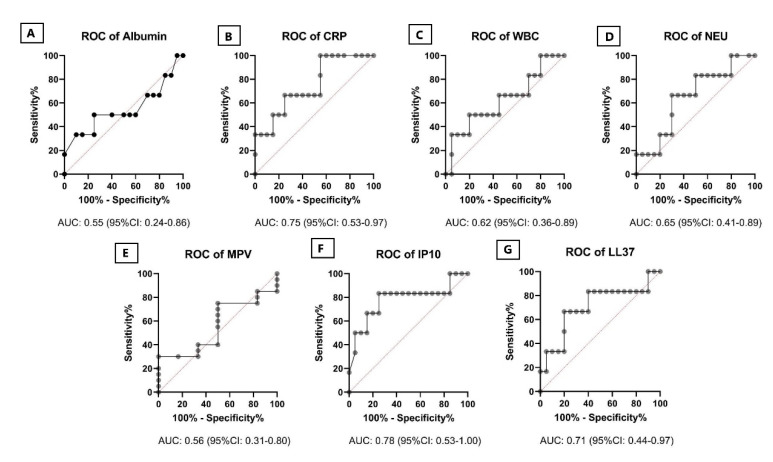
Receiver operating characteristic (ROC) curves for the individual biomarkers. The analysis of AUCs (area under the curve) for Albumin (**A**), CRP (**B**), WBC (**C**), NEU (**D**), MPV (**E**), IP10 (**F**) and LL37 (**G**). Black lines with dots indicate ROC curves. The AUC, followed by its 95% CI in brackets, was also illustrated over the bottom side of each figure. Abbreviations: CRP: C-reactive protein; WBC: white blood cell count; NEU: neutrophils; MPV: mean platelet volume; IP-10: interferon gamma inducible protein 10 and LL37: human cathelicidin peptide; ROC, receiver operating characteristic; CI, confidence interval; AUC, area under the curve.

**Figure 4:**
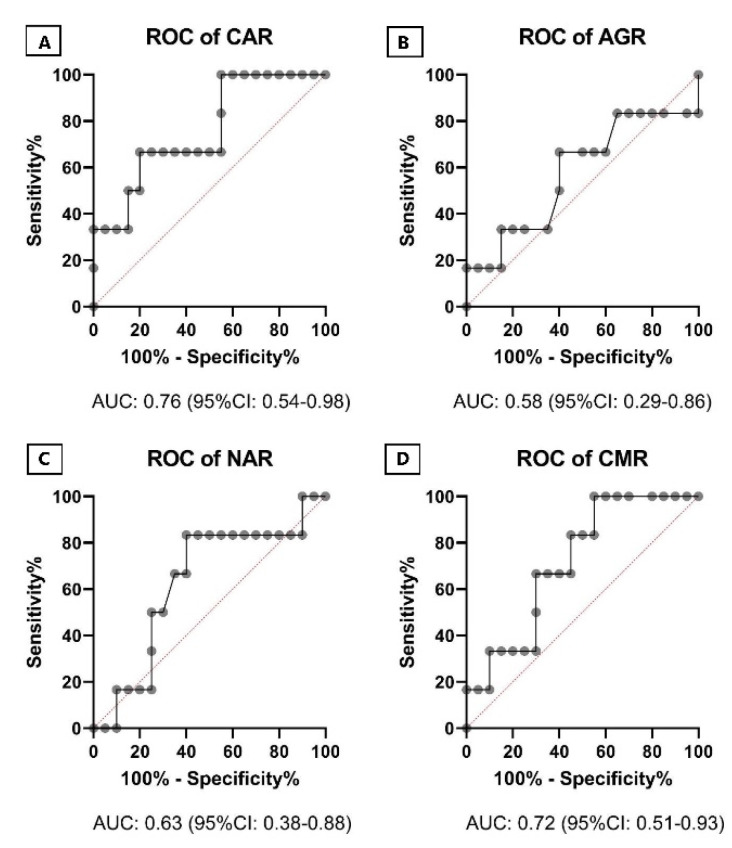
Receiver operating characteristic (ROC) curves for the ratios: CAR (**A**), AGR (**B**), NAR (**C**), CMR (**D**). Black lines with dots indicate ROC curves. The AUC, followed by its 95% CI in brackets, was also illustrated over the bottom side of each figure. Abbreviations: CAR: C-reactive protein (CRP) to albumin ratio; AGR: albumin to globulin ratio; NAR: neutrophil to albumin ratio; CMR: C-reactive protein to MPV ratio; ROC, receiver operating characteristic; CI, confidence interval; AUC, area under the curve.

ROC curve was used to evaluate the diagnostic value of single markers and ratios CRP, WBC, NEU, MPV, IP-10, LL-37, CAR, AGR, NAR and CMR. As shown in Figure 3, IP-10 showed the best AUC (0.78, 95% CI: 0.53–1.00) for the prediction of CULTURE (culture conversion) after two months of treatment. In the ROC curve analysis, with AUC greater than 0.7, CRP, LL-37, CAR and CMR could be considered good for prediction of CULTURE after treatment, as shown in Figure 3 and Figure 4.

**Figure 5:**
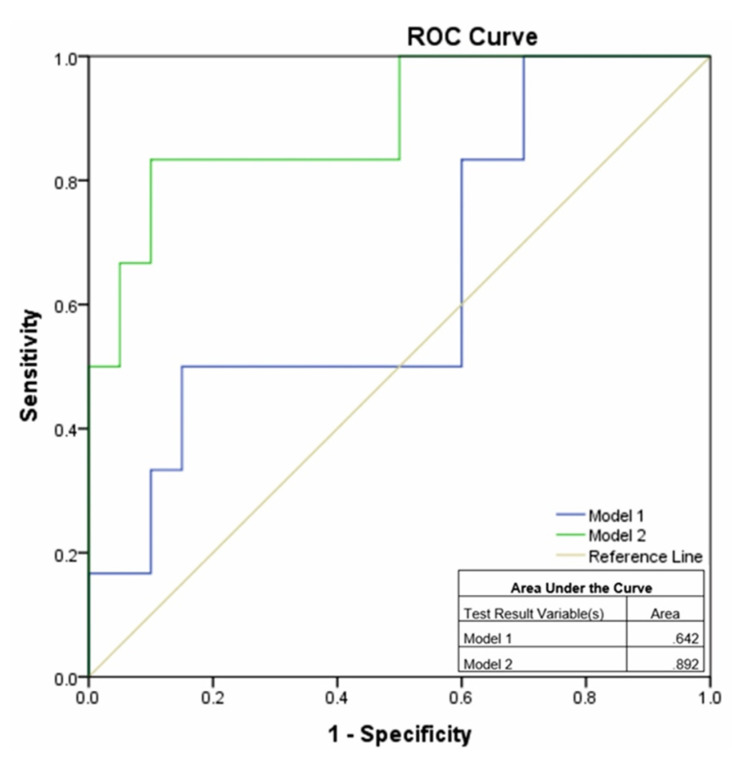
The area under the curve for the two models calculated from the logistic regression with best AUC. Abbreviations: AUC: area under the curve; ROC: receiver operating characteristic; Models 1–2 as represented in Table 5.

The logistic regression analysis showed the best models for combined parameters (Table 5). Model 2, containing a combination of seven biomarkers, presented a greater ability to predict CULTURE (culture conversion) after 60 days of anti-TB treatment (AUC 0.892; 95% CI: 0.732–1.0) than Model 1, as illustrated in Figure 5.

Use of a composite model of biomarkers improved the performance of prediction of culture-negative TB considering the AUC value close to 0.9 that indicates an excellent prediction efficacy.

The best model is Model 2 and the equation of prediction of the culture is: logit (*p*) = − 3.8 − 0.5 * WBC − 5.79 * NAR + 1.76 * NEU − 0.08 * CAR − 1.2 * CMR + 0.31 * CRP + 0.01 * IP10 where *p* is the probability of obtaining a negative culture after two months of treatment.

## 3. Discussion

To date, various inflammatory parameters have been used to evaluate the inflammatory process during mycobacterial infection, knowing that the inflammatory status has a very important role in the pathogenesis of TB [42]. Higher levels of some inflammatory markers used as prognostic indicators have been associated with unfavorable clinical outcomes [37]. White blood cell count, neutrophils, platelets, ESR, albumin, fibrinogen, globulin and C-reactive protein are the most commonly used laboratory markers to assess the inflammatory process [16,19]. In addition to these inflammatory biomarkers, interferon gamma-induced protein (IP-10), interferon gamma (INF-γ) or interleukin 6 (IL-6) have been used for monitoring therapy efficacy [43].

It has been demonstrated that other parameters, including antimicrobial peptides, such as LL-37, play an essential role in regulation of innate and adaptive immunity, influencing the activity of different cells which in turn intensify the course of inflammation [44,45].

Monitoring inflammatory status may be useful to predict anti-TB treatment outcomes and combining different immunological biomarkers could increase the predicting accuracy of treatment outcome [46].

As previously demonstrated, lower WBC and NEU values significantly correlated with negative sputum conversion at two months of treatment [47,48,49]. Our results are in line with these findings indicating that the decrease in WBC values in patients that turned negative culture at T2 is compatible with a more TB favorable outcome [14]. This contrasts with other studies which reported no statistical difference on WBC count after the intensive phase of treatment [50,51]. Thus, the persisting higher levels of WBC counts that are mostly reflective of neutrophil counts, in our patients that remained culture positive at T2, could indicate an ongoing infection and inflammation, as suggested by Srivastava et al. in their study [52]. Our results support the idea that the neutrophils play an important role in the inflammation associated with TB status, as indicated by other reports [53].

Furthermore, the decline in the NEU values was in turn reflected in a lower neutrophil/albumin ratio (NAR) in our patients that turned negative culture at T2, which could be explained either by lower neutrophil counts or higher albumin levels after the two months of therapy. In addition, we observed a highly significant positive correlation between NAR value and NEU counts.

Recently, NAR has been investigated as predictor of clinical outcomes in patients with cancer or other pathologies associated with an enhanced inflammatory status, but to the best of our knowledge no study has evaluated its role in monitoring anti-TB treatment [54,55]. In the present study, there was a more significant difference between patients that turned negative culture after two months of treatment and the same patients before treatment initiation in regard to the NAR value than the NEU counts, although the use of NAR, as an integrated ratio of neutrophil counts and albumin level, was not more predictive for culture status in the ROC analysis.

Albumin and globulin, the two main fractions of the serum protein, have proven to be critical markers linked to inflammation and infection [56]. To date, there is a growing evidence showing that high albumin levels or low globulin levels are associated with a better prognosis in patients with various types of cancer [57,58], including the chronic infectious TB disease [59]. A low albumin level is also associated with malnutrition or malabsorption, thus the higher levels of serum globulins generated by the accumulation of immunoglobulins and acute phase proteins are a better indicator of a more severe degree of inflammatory response [60]. As compared to positive culture TB patients at T2, TB patients that turned negative culture did not exhibit significantly higher levels of albumin or AGR. The lack of statistical significance regarding the normalization of these biomarkers could be explained by the small number of samples included in our study or by other factors, such as nutritional status.

In this study, alongside albumin, as an acute phase protein that responds to the systemic inflammation, we also focused to C-reactive protein (CRP), C-reactive protein to albumin ratio (CAR) and C-reactive protein to mean platelet volume (CMR), which have been studied before but not in patients with PTB in order to monitor treatment response in patients with active TB.

In the current study, we observed a markedly lower C-reactive protein level in patients that turned negative culture after two months of treatment compared to the patients that remained culture positive. Additionally, when we compared the subtracted CRP median values amongst the patients with positive culture versus the patients with negative culture, we noticed significantly decreased levels of CRP. These findings are in accordance with previous observations that found that C-reactive protein level has a large change with treatment and could be predictor for culture status [37]. Moreover, in our study, CRP to albumin ratio (CAR) has been found to be significantly lower in patients that turned negative culture. Studies have demonstrated that the combination of these two biomarkers as CRP to albumin ratio (CAR) is a better prognostic indicator than CRP or albumin alone [61]. Consistent with previous reports, we also found that AUC of CAR was higher than that of both CRP and albumin for predicting culture status and monitoring treatment response. In addition, the CRP presented a statistically significant correlation with CAR and CMR values in the Spearman correlation analysis, which are both correlated with the evolution of CRP value after anti-TB treatment [23].

We also investigated whether C-reactive protein to MPV ratio (CMR) is predictive for culture status. Mean platelet volume is another common inflammatory biomarker that is measured during a routine automatic whole blood count. MPV was well-documented alone or in relation to other inflammatory markers, such as CRP and ESR (erythrocyte sedimentation rate) in some chronic inflammatory disease to monitor the response to anti-inflammatory treatment [62,63]. Tozkoparan et al. observed that the MPV decreased significantly with anti-TB treatment [64], while others found similar values between patients with PTB and healthy subjects [65].

This is the first study to explore the predictive value of CMR in PTB patients. Our results found no statistical difference between the patients that turned negative culture after two months of treatment and the same patients that remained culture positive regarding both MPV and CMR value. Interestingly, we observed that AUC for CMR could be a good predictor of culture status, but these results must be confirmed by other studies. 

Many studies have evaluated the prediction potential of CRP for treatment outcomes in PTB patients in combination with different inflammatory biomarkers, such as the pro-inflammatory chemokine, cytokine, or antimicrobial peptide [65,66]. Interferon gamma (IFN-g)-inducible protein 10 (IP-10) is a pro-inflammatory chemokine that has been evaluated for the diagnostic potential in TB, but the results are varied [24,66,67]. Our study revealed significantly lower IP-10 values among the patients that turned negative culture at T2 compared to the patients which remained culture positive at T2, and these findings could be consistent with the reduction of any inflammatory state. Moreover, there was also a significant reduction of IP-10 when comparing the subtracted biomarkers’ median values or ratios from median values or ratios at T0 amongst the patients with positive culture versus the patients with negative culture. Here, it should be noted that IP-10 presented the greater ability to predict culture status after 60 days of anti-TB treatment as an individual marker, which confirms the results showed by previous studies [68,69,70].

CRP and IP-10 have been mostly evaluated in combination to determine if they can be used to increase the performance of differential diagnosis in TB [71]. Similar to other findings, that identified elevated concentrations of soluble IP-10 in plasma, serum or urine from PTB patients [72,73,74], this study found a declining tendency of IP-10 levels in patients that turned culture negative after anti-TB drug therapy compared to the levels at the time of initiation of treatment. Thus, the lack of treatment response could be associated with persistent high IP-10 levels.

In addition to IP-10, the antimicrobial peptide cathelicidin (LL-37) has been taken into consideration in the present study. LL-37 is an endogenous antimicrobial peptide involved in the innate immune response that is produced by various immune cells, such as neutrophils, monocytes, or T cells [20,22,45,75]. The role of LL-37 in the pathogenesis of TB has been previously reported [76,77] and besides its antimicrobial activity, LL-37 also has various effects including pro-inflammatory or anti-inflammatory functions [78]. Contrary to other studies [79], we noticed no significant changes in LL-37 levels between the initiation and completion of treatment in our PTB patients, which could be a result of the design of the experiment.

Various determinants have been found to be associated with culture non-conversion after the intensive phase of treatment, including male sex, older age, current active smoking, presence of hemoptysis or lung cavitation [80,81,82]. All the six patients with culture non-conversion were active smokers during the treatment, three of them presented hemoptysis, two presented cavitation and all of them had a history of alcohol abuse. Thus, the smoking habits, the impact of a prior alcohol abuse, hemoptysis or cavitation could be the main determinants of the delayed culture conversion in our patients, considering that sex and age were not significantly associated with culture status. Non-conversion of sputum culture has been found to be associated with hemoptysis, although the findings are contradictory [83,84]. Probably smoking cessation and resolving of hemoptysis would have resulted in a favorable outcome of these patients.

In the present work, culture negativity was significantly predicted in logistic regression analyses in a model consisting of seven predictors, WBC, NAR, NEU, CAR, CMR, CRP and IP10. These results could suggest that a composite model of biomarkers has a higher clinical accuracy than individual markers in predicting the treatment outcomes. Moreover, in comparison to individual markers, the AUC values of the composite model significantly improved prediction efficacy, from a good prediction efficacy to an excellent prediction efficacy of culture negativity. Of note, all predictors evaluated in the combined model presented declining values after anti-TB therapy as presented before.

This study has several limitations. Our study was a prospective pilot study with a low number of samples, thus large cohort studies of multicenter design should be conducted to confirm our results. The sample size group was limited by the restrictions imposed during the pandemic period of COVID-19, which limited the access to the hospital. Even if our study focused on the treatment outcomes, the inclusion of control groups would have been valuable. The sociodemographic data was limited to the patients’ cards containing the information. The collection of TB treatment outcome data was difficult after the intensive phase of treatment due to the voluntary reporting.

Because we only registered data after 60 days of anti-TB therapy, we were not able to assess the role of these biomarkers in predicting the treatment outcome after the four-month continuation phase. Molecular drug susceptibility testing was not assessed in this study. Few limitations such as contamination and variable turnaround time could be associated when using the month two culture as a predictive marker for treatment outcome.

Although the biomarkers included in our study reflect systemic inflammation, further prospective studies with various combinations of inflammatory factors are required in order to better predict or monitor the treatment outcomes in PTB patients. We did not evaluate other inflammatory markers such as ESR, fibrinogen, procalcitonin, interferon gamma (INF-γ) or interleukin 6 (IL-6) in combined models with our predictors. Further studies should be performed to compare the relative predictive value of these markers in composite models.

Despite these limitations, the combination of inflammatory biomarkers, rather than individual marker, presented in this study could have a great potential in predicting the treatment outcome. According to our results, CAR might be considered as a biomarker for monitoring anti-TB treatment. In addition, the highest significant trends and lower levels of CRP and IP-10 were observed in two-month treated TB patients, indicating the predictive value of these inflammatory markers for treatment outcome.

The main findings of our study were that a combination of inflammatory markers significantly increases the sensitivity and specificity compared to an individual marker model. The optimal combination of inflammatory factors will be determined by further large and prospective studies.

## 4. Materials and Methods

### 4.1. Study Subjects

We conducted a pilot prospective study of all newly diagnosed cases of PTB at Leamna Pneumophtisiology Hospital, Dolj County, Romania between June 2019, and March 2020. The entire protocol of this study was approved by the Ethics Committee from the University of Medicine and Pharmacy of Craiova, in compliance with the Declaration of Helsinki and its amendments (No. 5/17.01.2019).

A questionnaire was used by trained researchers to collect demographic data including age, gender, education, occupational status, alcohol consumption or tobacco use (Table 1). Individuals aged less than 18 years old or with extrapulmonary TB, previous TB history or death during antibiotic chemotherapy were excluded from the study. In addition, patients who had chronic illness, such as infectious disease, hypertension, hematological or autoimmune disease, diabetes mellitus, malignant conditions, hepatic or renal insufficiency, HIV infection or other comorbidities that can interfere with hematological and biochemical values of the analyzed parameters were also excluded from the study. Other exclusion criteria were as follows: patients taking drugs such as steroids or nonsteroidal anti-inflammatory drugs, patients who interrupted the treatment or patients whose data was missing.

All patients were diagnosed with TB based on extensive clinical evaluation, chest X-rays which were independently interpreted, culture of mycobacterium, and routine hematological and biochemical assays. All patients were subjected to drug a sensitivity test (DST). In patients with inconclusive chest X-rays, computer tomography (CT) was performed. As the initial diagnosis, sputum smear microscopy on up to three sputum samples was performed using auramine-rhodamine and Ziehl-Neelsen stainings. Mycobacterium cultures were performed on Löwenstein–Jensen (LJ) culture medium at the time of enrollment (T0) and after two months of treatment (T2) (data shown in the Supplementary File, Table S1).

Conventional drug susceptibility testing was performed for the first line anti-TB drugs using the Lowenstein Jensen (LJ) proportional method. DST was performed for rifampicin, isoniazid, ethambutol and streptomycin.

After applying all exclusion criteria, a total of 26 adult newly PTB patients from 38 patients were enrolled in our pilot prospective study and available for monitoring the treatment response after the two-month intensive phase.

All patients were treated in accordance with the national six-month regimen based on the WHO guidelines [4]. Following the two-month intensive phase, most of our patients were lost to follow-up after their discharge.

### 4.2. Blood Count Measurements and Biochemical Analysis

Three milliliters of venous blood were collected in tubes containing EDTA and five milliliters of venous blood were collected in tubes without anticoagulant at the first day of admission (T0) and discharge, after the two-month intensive phase (T2).

The blood count measurement was under strict quality procedure comprising twice-daily high, normal and low internal quality control (IQC), monthly external quality controls (EQS) and annual quality controls. Measurement of WBC (white blood cells), neutrophils (NEU) and mean platelet volume (MPV) were performed by standard procedure on an Abacus 5 Analyzer with five-part diff (Diatron, Budapest, Hungary).

The blood collected without anticoagulant was immediately centrifuged (4000 rpm × 5 min) to obtain the serum. The serum biomarkers, total protein, CRP, and serum albumin were evaluated using the Architect c8000 analyzer (Abbot Laboratories, Illinois, USA). Globulin was calculated using the following equation: [serum total protein (g/L)–serum albumin (g/L)].

The C-reactive protein (CRP) to albumin ratio (CAR), albumin to globulin ratio (AGR), neutrophil to albumin ratio (NAR), and C-reactive protein to mean platelet volume (MPV) were derived from variables measured at the same time point, either T0 or T2.

### 4.3. ELISA Methods

#### 4.3.1. IP-10 (CXCL10)

Serum IP-10 levels were measured using an enzyme-linked immunosorbent assay kit, IP-10 (CXCL10) Human ELISA kit, Invitrogen, Carlsbad, CA, according to the manufacturer’s recommendations. Following centrifugation at 3500 rpm for 10 min, the serum samples were separated and stored at −80 °C until analysis. The IP-10 levels were measured before base treatment initiation and after two months. All the serum specimens were tested in duplicate. Briefly, 50 μL of samples were incubated in ELISA wells of microtiter strips coated with a monoclonal antibody specific for human IP-10. Following incubation at room temperature for 3 h and washings, a biotinylated polyclonal secondary antibody to human IP-10 was added. After washing four times, streptavidin-HRP was added at room temperature for 30 min. After four washes, a stabilized chromogen was added and incubated for 30 min at room temperatures in the dark. The reaction was stopped and absorbance at 450 nm was measured within 2 h in an ELISA reader (Stat Fax 4200, Awareness Technology Inc, Palm City, FL, USA).

A standard curve was produced using freshly prepared serial dilutions of the kit’s reference standard from 500 pg/mL to 7.8 pg/mL.

#### 4.3.2. Human LL-37

A Human LL-37 (Antibacterial Protein LL-37) ELISA Kit (Elabscience, Houston, TX, USA) was used to measure LL-37 levels in serum according to the manufacturer’s instructions. The samples were collected into serum separator tubes and centrifuged at 3500 rpm for 10 min. The serum samples were kept frozen at −80 °C until analysis. The LL-37 levels were measured before treatment initiation and after the completion of two months of treatment. LL-37 measurements were done in duplicate. Serum samples (100 μL) were added to ELISA wells pre-coated with an antibody specific to human LL-37 and incubated for 90 min at 37 °C. Immediately, 100 μL of biotinylated detection antibody specific for human LL-37 was added and incubated 60 min at 37 °C. After washing 4 times, 100 μL of streptavidin-horseradish peroxidase conjugate (HRP) solution was added and after 30 min at 37 °C, following 5 washes, 90 μL of TMB substrate was added to each well. The enzyme-substrate reaction was terminated by the addition of stop solution and the absorbance were read at 450 nm within 30 min using the same ELISA plate reader (Stat Fax 4200, Awareness Technology Inc.).

A standard curve was produced using freshly prepared serial dilutions of the kit’s reference standard from 50 ng/mL to 1.56 ng/mL.

### 4.4. Statistical Analysis

Statistical analysis was carried out with GraphPad Prism 9.0.0 (GraphPad Software, San Diego, CA, USA) and R (version 4.0.3, GNU General Public Licence). Continuous variables were given as mean (standard deviation) or median (interquartile range), while categorical variables were expressed as the number of subjects (n) and the percentage value (%). Because of the small number of patients, nonparametric Mann–Whitney U test was applied to compare the values of biochemical markers between patients stratified according to culture status after two months of TB treatment or between patients at basis vs. patients after two months of treatment.

Frequencies of categorical variables were compared using Fisher’s exact test. The results were considered statistically significant at the 5% level (two-tailed).

Spearman’s correlation test was carried out to analyze the relationship between variables. The R corrplot package was used to construct a correlogram with hierarchical clustering to display the strength and direction of all biomarker correlations.

Binary logistic regression model was conducted to predict the negative culture after two months of TB treatment and assess whether the biomarkers that reached statistical significance could delineate a correlation with the culture status. The logistic regression pointed out the major variables as predictors for culture following two months of anti-TB treatment.

Receiver operation characteristic (ROC) curve and the area under the ROC curve (AUC) with 95% confidence intervals (CI) were performed for each parameter and were calculated by time-dependent receiver operating characteristic curves as prognostic factors for treatment outcome in tuberculosis. 

AUC was also calculated to measure the prognostic accuracy for models containing marker combinations and each potential predictor of treatment outcome. It was expected that AUC to would be between 0.5, indicating no discriminative ability, and 1.0, indicating highest detection accuracy. Results were considered significant if the 95% CI of AUC exceeded 0.7.

## Figures and Tables

**Table 1:** Characteristics of the total sample, participants with positive culture, and with negative culture.

Characteristics	Total (n = 26)	Positive Culture (n = 6)	Negative Culture (n = 20)	*p*-Value
Age (years)	47.85 ± 8.98	48.17 ± 9.5	47.75 ± 9.1	0.9649
Gender Female Male	3 (12%) 23 (88%)	1 (3.8%) 5 (19.2%)	2 (7.7%) 18 (69.2%)	>0.99
Living environment Urban Rural	6 (24%) 20 (76%)	1 (3.8%) 5 (19.2%)	5 (19.2%) 15 (57.7%)	>0.99
Educational level Low Middle	2 (8%) 24 (92%)	1 (3.8%) 5 (19.2%)	1 (3.8%) 19 (73.1%)	0.4154
Smoker Yes No	18 (69%) 8 (31%)	4 (15.4%) 2 (7.7%)	14 (53.8%) 6 (23.1%)	>0.99
Alcohol Yes No	3 (12%) 23 (88%)	0 6 (23.1%)	3 (11.5%) 17 (65.4%)	>0.99

Mean ± standard deviation is provided for continuous variables. Number of patients (percentages) is provided for categorical variables.

**Table 2:** Comparison of all biomarkers values before the initiation of anti-TB therapy and after completion of the intensive phase of treatment for patients that turned culture negative at T2.

Biomarkers	T0 (n = 20)	T2 (n = 20)	*p*-Value
Albumin (g/dL)	3.64 ± 0.54	3.94 ± 0.45	0.0596
CAR	12.87 ± 12.08	5.27 ± 5.22	0.0263 *
AGR	1.04 ± 0.27	1.11 ± 0.24	0.2336
NAR	2.10 ± 1.20	1.33 ± 0.54	0.0020 ***
CMR	7.08 ± 7.3	3.77 ± 3.92	0.0898
CRP (mg/L)	43.06 ± 38.68	19.18 ± 17.67	0.0268 *
WBC	10.20 ± 2.99	8.18 ± 1.91	0.0181 *
NEU (10^3^/uL)	7.28 ± 2.96	5.10 ± 1.75	0.0061 *
MPV (fL)	6.62 ± 1.21	6.02 ± 1.49	0.1510
IP10	382.9 ± 97.26	294.1 ± 108.4	0.0080 **
LL37	42.26 ± 30.98	34.10 ± 27.34	0.1918

Abbreviations: CAR: C-reactive protein (CRP) to albumin ratio; AGR: albumin to globulin ratio; NAR: neutrophil to albumin ratio; CMR: C-reactive protein to MPV ratio; CRP: C-reactive protein; WBC: white blood cell count; NEU: neutrophils; MPV: mean platelet volume; IP-10: interferon gamma inducible protein 10 and LL37: human cathelicidin peptide. Bold indicates significantly difference between T0 and T2. *, *p* < 0.05; **, *p* < 0.01, ***, *p* < 0.001. Mean ± standard deviation is provided for continuous variables.

**Table 3:** Comparison of the subtracted values (values T2–values T0) of biomarkers between the patients with positive culture and the patients with negative culture.

T2-T0	Positive Culture (n = 6)	Negative Culture (n = 20)	*p*-Value
Albumin (g/dL)	0.65 ± 0.6	0.29 ± 0.28	0.1649
CAR	−7.1 ± 15.69	−7.6 ± 8.83	0.2425
AGR	0.2 ± 0.28	0.07 ± 0.12	0.5620
NAR	−0.64 ± 0.57	−0.77 ± 1.01	0.8937
CMR	−1.89 ± 5.77	−3.32 ± 5.69	0.2816
CRP (mg/L)	−12.61 ± 38.99	−23.88 ± 28.88	0.0459 *
WBC (10^9^/L)	−0.9 ± 1.06	−2.02 ± 2.34	0.2681
NEU (10^9^/L)	−0.89 ± 1.03	−2.18 ± 2.49	0.3006
MPV (fL)	−0.13 ± 0.27	−0.6 ± 1.4	0.2362
IP10 (pg/mL)	23.42 ± 40.65	−88.83 ± 77.5	0.0006 ***
LL37 (ng/mL)	1.68 ± 12.9	−8.17 ± 22.84	0.0702

Abbreviations: CAR: C-reactive protein (CRP) to albumin ratio; AGR: albumin to globulin ratio; NAR: neutrophil to albumin ratio; CMR: C-reactive protein to MPV ratio; CRP: C-reactive protein; WBC: white blood cell count; NEU: neutrophils; MPV: mean platelet volume; IP-10: interferon gamma inducible protein 10 and LL37: human cathelicidin peptide. Bold indicates significantly difference between the patients with positive culture and the patients with negative culture. *, *p* < 0.05; ***, *p* < 0.001. Mean ± standard deviation is provided for continuous variables.

**Table 4:** Matrix of correlation coefficients among the biomarkers and their ratios.

	Age	Albumin	CAR	AGR	NAR	CMR	CRP	WBC	NEU	MPV	IP10	LL-37
Age	1	−0.31	0.22	0.01	0.18	0.25	0.21	0.08	0.04	-0.23	0.10	−0.27
Albumin		1	**−0.71**	**0.73**	**−0.52**	**−0.63**	**−0.67**	−0.17	−0.29	0.23	**−0.69**	0.39
CAR			1	−0.6	**0.7**	**0.96**	**0.99**	**0.48**	**0.56**	**−0.40**	**0.77**	−0.13
AGR				1	**−0.51**	**−0.59**	**−0.58**	**−0.30**	−0.37	0.32	**−0.51**	**0.50**
NAR					1	**0.72**	**0.68**	**0.88**	**0.95**	**−0.44**	**0.66**	**−0.39**
CMR						1	**0.97**	**0.53**	**0.6**	**−0.58**	**0.71**	−0.24
CRP							1	**0.48**	**0.55**	**−0.42**	**0.76**	−0.12
WBC								1	**0.93**	−0.32	0.41	−0.25
NEU									1	**−0.40**	0.53	−0.27
MPV										1	−0.20	**0.59**
IP10											1	−0.14
LL-37												1

Bold indicates significantly correlated variables, *p* < 0.05.

**Table 5:** The area under the curve for the composite models of biomarkers.

Model	Predictors	AUC, 95% CI	*p*-Value
Model 1	Albumin, AGR, LL37, MPV	0.642 0.381–0.902	0.301
Model 2	WBC, NAR, NEU, CAR, CMR, CRP, IP10	0.892 0.732–1.0	**0.004**

Bold indicates significantly correlated variables, *p* < 0.05.

## Data Availability

The data presented in this study are available in Supplementary Materials, Table S1: The Laboratory Data.

## Supplementary Materials

The following are available online at https://www.mdpi.com/article/10.3390/pathogens10070789/s1, Table S1: The laboratory data.
